# Exploring Maternal Challenges: A Pilot Study of Pain, Fatigue, and Anxiety in Newborn Care Within Rooming-in Settings

**DOI:** 10.3390/jcm14010207

**Published:** 2025-01-02

**Authors:** Prokopowicz Anna, Tułacz Kinga, Jabłońska Anna, Bagłaj Maciej, Rozensztrauch Anna

**Affiliations:** 1 Division of Fundamentals of Midwifery, Department of Midwifery, Wroclaw Medical University, 50-367 Wroclaw, Poland; anna.prokopowicz@umw.edu.pl (P.A.); anna.jablonska@umw.edu.pl (T.K.); kinga.tulacz@umw.edu.pl (J.A.); 2 Department of Pediatrics and Coordinated Child Care, Wroclaw Medical University, 50-367 Wroclaw, Poland; baglaj.maciej@umw.edu.pl

**Keywords:** anxiety, pain, fatigue, rooming-in, maternity, maternal care

## Abstract

**Background:** In the rooming-in system, mothers and their healthy newborns stay together for 24 h a day; however, many women in the early postpartum period often find it challenging to balance their recovery from childbirth with the demands of caring for their newborns. This study aims to investigate the need for postpartum women to entrust their newborns to medical staff for care, and the relationship of this need with perceived pain, fatigue, and anxiety. **Methods:** The study uses the Need to Entrust a Newborn under the Care of the Staff (NEN) scale and the Numerical Rating Scale (NRS) to assess participants’ levels of pain, fatigue and anxiety. These scales were chosen to provide a comprehensive assessment of participants’ needs and experiences. **Results:** The results of the study reveal that fatigue levels among study participants were significantly high, with an Me of 7.0 (IQR = 4.0), exceeding the reportable levels of both pain (Me = 6.0, IQR = 5.0) and anxiety (Me = 5.0, IQR = 6.0) The need for support during the day and at night was at a similar level and strongly correlated (rho = 0.723; *p* < 0.001). Pain levels showed a significant positive correlation with the need to entrust the newborn both during the day (rho = 0.296; *p* < 0.001) and at night (rho = 0.332; *p* < 0.001). During the daytime, the correlation of fatigue with the need for staff support was rho = 0.423 (*p* < 0.001), while overnight, this increased to rho = 0.485 (*p* < 0.001). Anxiety significantly correlated with the need for staff support, both during daytime (rho = 0.422; *p* < 0.001) and overnight (rho = 0.431; *p* < 0.001). Multiparas reported significantly lower results of anxiety (U(Z) = −13.224, *p* < 0.001). **Conclusions:** The need to entrust newborns to the care of maternity rooming-in staff is strong but is often unmet in many facilities. Further research should be conducted to explore solutions, and plan future actions to alleviate the burdens on postpartum women and facilitate their recovery.

## 1. Introduction

Perinatal care has undergone a tremendous transformation over the past century. Women increasingly give birth in hospitals, leading to a gradual decline in home births. The system adopted at the beginning of this change involved separating the mother from the child and placing the newborn in “newborn nurseries.” Newborns were provided with care, while mothers were given the opportunity for peaceful sleep and rest [1]. At the end of the 20th century, hospitals started implementing the practice of rooming-in, which, according to the World Health Organization and the United Nations International Children’s Emergency Fund (UNICEF), is a hospital practice where mothers and their healthy newborns remain together in the same room, 24 h a day, from the moment they are admitted to the room after birth. The rooming-in system promotes the initiation of early breastfeeding and encourages bonding between the mother and the baby, which can positively influence postpartum recovery and overall family wellbeing [2,3]. Mothers in the rooming-in system produce larger amounts of milk, breastfeed for longer periods, and are more likely to practice exclusive breastfeeding [4,5]. Additionally, while this facilitates the early identification of a baby’s needs, it can also pose challenges, particularly for first-time mothers or those recovering from childbirth. In the current rooming-in system, women face challenges in simultaneously “recovering” from childbirth and caring for their infants. Postpartum women often feel overwhelmed, due to a lack of experience in newborn care. This is compounded by fatigue from labor, sleep deprivation, and mental health conditions [6,7]. These negative feelings represent negative birth experiences and are correlated with the occurrence of postpartum depression and post-traumatic stress disorder. Furthermore, fatigue and poor sleep quality is linked to an increased risk of postnatal depression, can impair mother–child interactions and impede the child’s emotional and cognitive development [8]. Most authors indicate that women who have vaginal births are more fatigued than those who have cesarean sections [9,10]. Therefore, while the rooming-in system offers significant advantages, it is crucial to address maternal fatigue to fully realize its potential benefits. Providing adequate support and opportunities for rest can help mitigate the negative effects of fatigue, enhancing both maternal wellbeing and infant care. The pain and anxiety often experienced after delivery can further intensify these challenges, making it more difficult for mothers to physically and emotionally recover. Providing sufficient support and opportunities for rest is essential to relieve these negative effects, promote maternal wellbeing and enable optimal baby care [11].

Considering the impact of pain, fatigue, and anxiety on mothers’ wellbeing, our aim was to assess women’s need to temporarily entrust the care of their newborns to healthcare providers during postpartum recovery. Additionally, our study aimed to examine the relationship between this need and factors such as pain, fatigue, and anxiety.

## 2. Materials and Methods

### 2.1. Sampling Method

The study employed a snowball sampling method, facilitated via an online platform. This method was chosen due to its efficiency in reaching a dispersed population and its ability to leverage social networks for broad participant recruitment. Snowball sampling began with initial participants, who were identified and invited to complete the survey. These initial participants were then encouraged to share the questionnaire link with others in their network who met the inclusion criteria. This approach allowed the study to efficiently gather a large and diverse sample of eligible participants. Ethical approval for the study was obtained from the bioethics committee (Approval No. KB–292/2022).

### 2.2. Inclusion and Exclusion Criteria

To ensure a focused and relevant dataset, strict inclusion and exclusion criteria were established:

### 2.3. Inclusion Criteria

Participants were adult women (aged 18 or older) residing in Poland.

Participants had given birth after 1 January 2019.

Participants stayed with their newborn in a rooming-in maternity unit during the postpartum period.

Participants were required to provide informed consent.

Participants demonstrated fluency in both spoken and written Polish to ensure accurate and reliable questionnaire responses.

Participants reflected on their psychophysical state during the first three days of the postpartum period after their most recent delivery.

Participants were able to access and complete an online questionnaire independently.

### 2.4. Exclusion Criteria

Questionnaires were excluded if they were incomplete, inconsistent, or failed to meet the inclusion criteria.

Inconsistencies included contradictory or implausible responses identified during the review process.

Participants who did not stay in a rooming-in maternity unit after delivery were excluded.

Participants who provided responses outside the study timeframe or in a language other than Polish were excluded.

### 2.5. Data Collection and Quality Control

Data collection was conducted between April 2022 and March 2023. Eligible participants were invited to retrospectively reflect on their psychophysical state during the first three days of the postpartum period following their most recent delivery. A total of 1492 questionnaires were submitted during the study period.

To ensure data quality and reliability, all questionnaires underwent a rigorous review process. A panel of experts in gynecology, midwifery, and research methodology reviewed each questionnaire twice. This meticulous validation process aimed to identify and exclude incomplete, inconsistent, or improperly filled questionnaires. As a result, 82 questionnaires were excluded, leaving a final dataset of 1410 fully completed and verified responses.

### 2.6. Statistical Analysis

The validated dataset was subjected to comprehensive statistical analysis to derive meaningful insights. Descriptive statistics, including frequencies, percentages, means, and standard deviations, were used to summarize the demographic and baseline characteristics of the participants. Inferential statistics were applied to identify significant patterns and relationships within the data.

### 2.7. Correlation Analysis

Statistical analysis was conducted using IBM SPSS Statistics 29 (IBM Corp., Armonk, NY, USA). The obtained statistical values indicated that most variables significantly deviated from a normal distribution; therefore, non-parametric tests were used for most analyses. The relationship between variables was assessed using Spearman’s rho correlation. The Mann–Whitney U test was employed for comparisons between independent groups. A significance level of *p* < 0.05 was adopted for all measurements.

### 2.8. Research Tools

To characterize the study group, a self-authored questionnaire was used (SAQ) containing questions about sociodemographic and medical data. Participants were then stratified by age, mode of delivery, and between primiparous and multiparous women. The need to place a newborn in the care of staff was assessed the Need to Entrust a Newborn under the Care of the Staff (NEN) scale [12].

### 2.9. The Need to Entrust a Newborn Under the Care of the Staff (NEN) Scale

This questionnaire focused on women’s needs during both daytime and nighttime. The questions were as follows:

Considering my psychophysical state after childbirth, I felt the need to entrust my newborn to the care of staff for some time during the day (NEN-D).

Considering my psychophysical state after childbirth, I felt the need to entrust my newborn to the care of staff for some time at night (NEN-N).

Additionally, respondents were asked for their opinion on the mandatory option to temporarily transfer care of their newborn to medical staff in maternity wards. They responded to the question: “I believe that a woman after childbirth should have the option to periodically transfer care of her newborn to medical staff” (NEN-opinion). Respondents also answered the question: “During my stay in the hospital on the rooming-in ward, was I informed by the medical staff about the option to temporarily transfer my newborn’s care to them if I felt the need?” (NEN-information).

The responses were given on a 6-point Likert scale (1—“I strongly disagree”, 2—“I disagree”, 3—“I disagree a little”, 4—“I agree a little”, 5—“I agree”, and 6—“I strongly agree”). To illustrate the dichotomous tendency in the respondents’ answers, respondents who rated their need from 1 to 3 were grouped into the category of patients with a low need to entrust their newborn to the care of medical staff during the day/night (NEN-D_low and NEN-N_low), while responses rated from 4 to 6 were assigned to the group of patients indicating a high need to temporarily entrust their newborn’s care to medical staff (NEN-D_high and NEN-N_high). Similarly, the variables NEN-opinion and NEN-information were also dichotomized.

To measure pain, fatigue, and anxiety, a Numerical Rating Scale (NRS) from 0 to 10 was used (0 indicates no pain (NRS)/fatigue (NRS-F; fatigue)/anxiety (NRS-A; anxiety), while 10 represents the worst pain, fatigue, or anxiety one can imagine) [13,14,15,16]. The use of simple single-item scales appears to be the simplest form of diagnostic tool; however, they are still rarely utilized.

### 2.10. Rationale and Advantages of Using These Scales

The Need to Entrust a Newborn under the Care of the Staff (NEN) scale evaluates a mother’s emotional need to rely on medical staff for the care of her newborn. It captures the degree to which the mother feels dependent on professionals, and whether she trusts them to take care of her child, which may be influenced by factors such as physical health, emotional state, and confidence in her ability to care for the baby. The advantage of using the NEN scale is that it provides direct insight into the mother’s emotional needs and experiences during the postpartum period, helping healthcare providers understand when extra support may be necessary for the mother.

The Numerical Rating Scale (NRS) is a widely used tool to measure subjective experiences, particularly pain, fatigue, and anxiety. Participants are asked to rate the intensity of these feelings on a scale from 0 (no pain/fatigue/anxiety) to 10 (worst possible pain/fatigue/anxiety). Its simplicity and ease of use make it a popular choice in clinical settings, as it allows for quick assessment and facilitates data collection. It also provides quantifiable data that can be used to track changes in a patient’s condition over time.

By using both scales, a study can explore the relationship between a postpartum mother’s emotional need to entrust her newborn to medical staff (as measured by the NEN scale) and her physical and emotional distress (as measured by the NRS). This combination of scales provides a comprehensive view of a mother’s postpartum experience, linking both emotional and physical factors that may influence her decision to seek professional care for her newborn.

## 3. Results

### 3.1. Study Population

The study included 1410 women. The average age was 31 years (range: 18–44). In the study group, 42.6% (n = 600) of the patients had a cesarean section, while 57.4% (n = 810) gave birth via vaginal delivery. Among the respondents, 59.6% (n = 840) were primiparous, and 40.4% (n = 570) were multiparous. Most, 98.7, were in a relationship, 58.3 attended a birthing school, and a majority, 82.4, of them had a university degree.

### 3.2. Results for NRS and NEN Scales

The results of the study, highlighted in Table 1, reveal that fatigue levels among study participants were significantly high, with a median score of 7.0 (IQR = 4.0), exceeding the reportable levels of both pain (Me = 6.0, IQR = 5.0) and anxiety (Me = 5.0, IQR = 6.0) on the Numerical Rating Scale (NRS). These results indicated that fatigue was the most prominent physical and emotional challenging item reported by postpartum women. Moreover, the results revealed a strong demand to entrust their newborns to the staff of the maternity ward at night compared to during daytime. A total of 53.0% (n = 747) of participants indicated a high need for night-time support (NEN-N_high), while 47.0% (n = 663) indicated a lower need (NEN-N_low). In the daytime, by contrast, 46.1% (n = 650) indicated a high need (NEN-D_high) and 53.9% (n = 760) indicated a low need (NEN-D_low). These results highlight the perceived increased need for night-time support, when the fatigue and challenges of caring for a newborn may be enhanced.

More notably, 92.7% (n = 1307) of participants were strongly of the opinion that women in rooming-in maternity wards should have the right to have their newborns under the care of medical staff (NEN-opinion, Me = 6.0, IQR = 1.0). Despite this overwhelming demand for additional support, however, 65.5% (n = 923) of participants indicated that they had not been informed of this option by maternity staff (NEN-information, Me = 2.0, IQR = 3.0).

**Figure 1:**
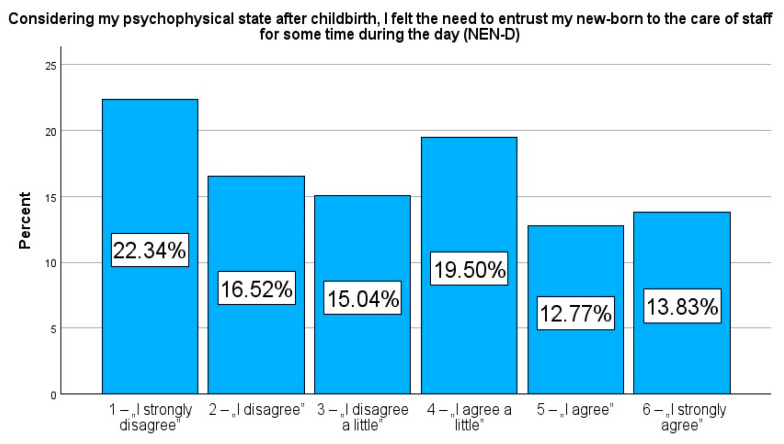
Percentage rates of the variables NEN-D.

**Figure 2:**
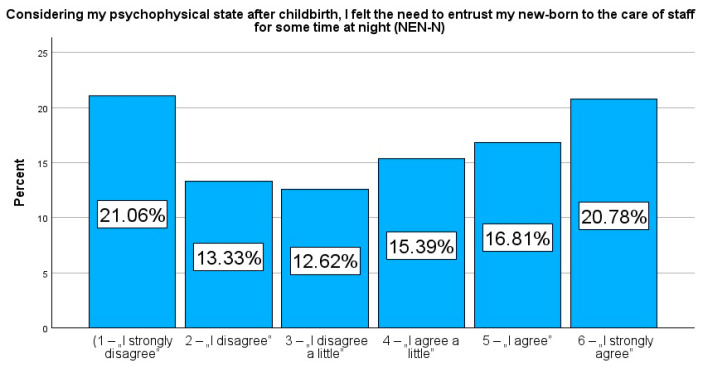
Percentage rates of the variables NEN-N.

**Figure 3:**
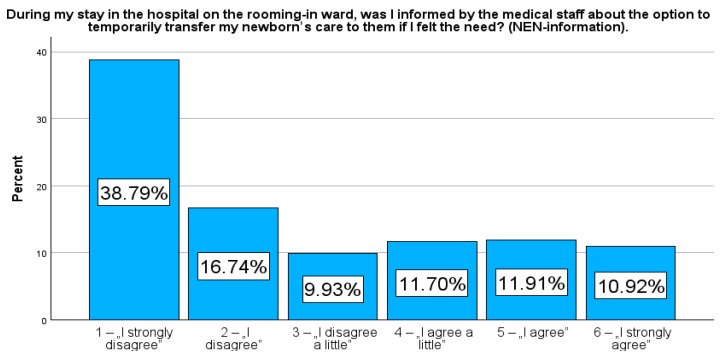
Percentage rates of the variables NEN-information.

**Figure 4:**
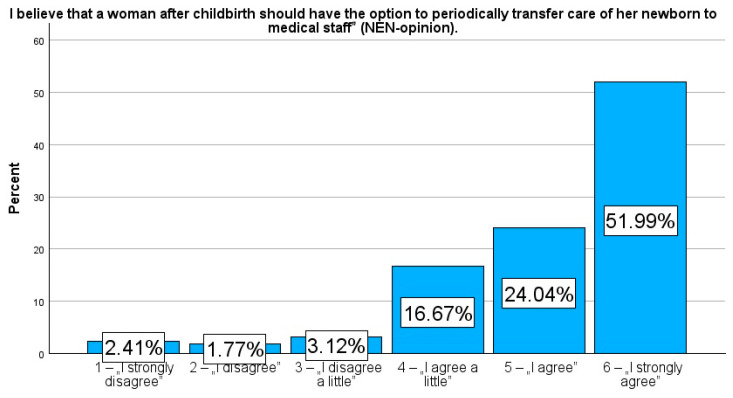
Percentage rates of the variables NEN-opinion.

Presenting the prevalence as a percentage allows a more detailed comparison of specific responses in relation to selected variables. In contrast to the median or mean, which only provide an averaged picture of the study population, frequency analysis allows a better understanding of the variety of variables and their specificities. Therefore, in addition, to strengthen the picture, these results are additionally presented in figures to emphasise the importance of frequency and facilitate their interpretation. Rates reflecting the percentage of variables, NEN-D, NEN-N, NEN-information, and NEN-opinion, are presented in Figure 1, Figure 2, Figure 3 and Figure 4.

### 3.3. Relationship Between the Need to Entrust the Newborn to the Care of Medical Staff and Pain, Anxiety, and Fatigue

Correlation coefficient analysis between NRS and NEN results revealed some significant findings, indicating strong correlations between the need for maternal support and levels of pain, fatigue, and anxiety. A strong positive correlation was found between the need to entrust the newborn to medical staff during the day and at night (rho = 0.723; *p* < 0.001), implying that, when women expressed a strong need for support during daytime, they expressed a similarly high need for nighttime support. This correlation emphasizes the consistent need for support at different times of the day, possibly due to ongoing challenges such as fatigue and discomfort.

However, subsequent analysis revealed that the need for support from staff (both during the day and at night) was significantly correlated with levels of pain, fatigue, and anxiety. For a case in point, pain levels showed a significant positive correlation with the need to entrust the newborn both during the day (rho = 0.296; *p* < 0.001) and at night (rho = 0.332; *p* < 0.001), indicating that women who experienced higher levels of pain were more likely to request help. Although the correlations of pain were moderate, they emphasize the impact of physical pain and discomfort on the need for additional maternal support. The fatigue level had the strongest correlations with the need for staff support. During daytime, the correlation of fatigue with the need for staff support was rho = 0.423 (*p* < 0.001), while overnight, it increased to rho = 0.485 (*p* < 0.001). These numbers strongly suggest that fatigue is a significant factor affecting mothers’ preferences for staff support, especially overnight, when exhaustion may be more evident after a full day of caring for the newborn. Anxiety was similarly significantly correlated with the need for staff support both during daytime (rho = 0.422; *p* < 0.001) and overnight (rho = 0.431; *p* < 0.001). These correlations indicated that higher levels of anxiety are related to a higher observed need to be temporarily entrusted with the care of the newborn by medical staff. This data is shown in Table 2.

### 3.4. Comparison by Mode of Delivery and Number of Births

Women who gave birth via cesarean section reported a higher need to entrust their newborn to the care of medical staff during the day and at night, and experienced higher levels of pain, fatigue, and anxiety compared to women who gave birth naturally. First-time mothers reported a higher need to entrust their newborns to the care of staff during the day and at night, and experienced higher levels of pain, fatigue, and anxiety compared to the group of multiparous women (Table 3). The analysis in Table 3, below, compares the results between the the two groups, the VD group (vaginal delivery) (n = 810) and the CC group (cesarean section) (n = 600), as a well as between primiparous (n = 840) and multiparous mothers (n = 570).

### 3.5. Results Overview NRS

Resulted in a statistical significant difference (U(Z) = −14.361, *p* < 0.001), indicating that mothers who had undergone a cesarean section reported higher levels of pain than those who had a vaginal delivery. Among primiparous women, the median score was 6.00 (Mrank = 732.27), while multiparous women reported a median score of 5.00 (Mrank = 666.06), also showing a significant difference (U(Z) = −3.013, *p* < 0.05). For fatigue, the median score in the VD group was 7.00 (Mrank = 652.13), compared to a median of 8.00 in the CC group (Mrank = 777.55), reflecting a significant difference (U(Z) = −5.770, *p* < 0.001). This suggests that mothers who experienced CC were more likely to experience fatigue than those who experienced vaginal delivery. The median fatigue score for primiparous women was 8.00 (Mrank = 764.46), while multiparous women had a lower median score of 7.00 (Mrank = 618.61), indicating a significant difference (U(Z) = −6.660, *p* < 0.001). There, the median anxiety score for the VD group was 4.00 (Mrank = 656.80), as compared to the CC group, which had a median score of 5.00 (Mrank = 771.25), which resulted in significant differences (U(Z) = −5.242, *p* < 0.001). This indicated that CC mothers experienced higher levels of anxiety.

### 3.6. Need to Entrust During the Day/Night

Multiparas reported significantly lower results of anxiety (U(Z) = −13.224, *p* < 0.001). For the NEN-D scale, the VD group had a median score of 3. 00 (Mrank = 648.75), while the CC group recorded a higher median score of 4.00 (Mrank = 782.11), indicating a significant difference (U(Z) = −6.178, *p* < 0.001). Thus, this implies that cc mothers experienced a greater need to entrust their newborns to medical staff during daytime. Primiparous mothers had a median score of 4.00 (Mrank = 749.60), indicating a significant difference (U(Z) = −6.00, *p* < 0.001). As for overnight need, the VD group reported a median of 3.00 (Mrank = 620.81) compared to CC mothers, who had a median of 4.50 (Mrank = 819.84), which was a statistically significant (U(Z) = −9.220, *p* < 0.001), indicating that cc mothers experienced a higher need for assistance overnight. In terms of nighttime, the median score for primiparous mothers was higher than for multiparous with a significant difference (U(Z) = −5.228, Mrank = 751.46, *p* < 0.001).

Table 4 presents regarding overall discomfort, as assessed by the Numerical Rating Scale (NRS), both the POV and NV groups scored a median of 6.00, with similar mean ranks (693.72 for POV and 714.02 for NV). Similarity indicated that there was no significant difference between these groups in perceived discomfort (U(Z) = −0.929 U(Z) = −0.929 U(Z) = −0.929). For the NEN-I (Negative Emotional Needs Index) comparison, both the high and low NEN-I groups also reported a median score of 6.00 on the NRS, which is interesting, although the mean rank was slightly lower in the high NEN-I group (678.53) compared to the low NEN-I group (719.73), which revealed statistical significance (U(Z) = −1.816 U(Z) = −1.816 U(Z) = −1.816). For fatigue (NRS-F), both POV and NV groups, respectively, reported the same Me score of 7.00, and the Mranks were nearly identical (708.65 for POV and 703.22 for NV), indicating that there was no significant difference in fatigue levels (U(Z) = −0.249 U(Z) = −0.249 U(Z) = −0.249). A significant difference between the high and low NEN-I groups appeared; however, while the high NEN-I group reported a Me score of 7.00 with a lower Mrank (621.05), the low NEN-I group had a higher median score of 8.00, and a mean rank of 750.06. This difference was statistically significant (U(Z) = −5.708, *p* < 0.01 U(Z) = −5.708, *p* < 0.01 U(Z) = −5.708, *p* < 0.01), implying that women in the low NEN-I group reported greater fatigue. Levels of anxiety (NRS-A) revealed a slight difference between the POV and NV groups. Although this difference was not statistically significant (U(Z) = −1.560 U(Z) = −1.560 U(Z) = −1.560), it suggests a trend toward slightly elevated levels of anxiety in the NV group. In the NEN-I comparison, a similar trend was observed: the high NEN-I group reported a median anxiety score of 4.00, compared to 5.00 for the low NEN-I group. This difference was statistically significant (U(Z) = −5.008, *p* < 0.01 U(Z) = −5.008, *p* < 0.01 U(Z) = −5.008, *p* < 0.01), indicating that women with lower NEN-I experienced higher levels of anxiety.

## 4. Discussion

In this study, we examined whether women in the early postpartum period, staying in a hospital under the rooming-in system, need to temporarily entrust their newborn to the care of medical staff, and the relationship of this need with the levels of pain, anxiety, and fatigue. The study was conducted in recognition of the need for critical need for appropriate care in the immediate postnatal period, as the early days after delivery frequently involve substantial challenges in caring for the newborn. The literature strongly suggests that an essential period is required to establish a mother–baby bond and initiate successful breastfeeding, both of which have long-term consequences for the health and wellbeing of both mother and baby [17,18].

The results showed that women reported a greater need to place their newborn under the care of maternity unit staff at night compared to during the day; however, a high need was expressed by almost half of the participants both during the day and at night (46.1% and 47.0%). Additionally, 92.7% of women expressed the opinion that they should have the right to receive support from maternity staff in caring for their newborns after birth; however, 65.5% of them were not even informed about such a possibility. The levels of pain, fatigue, and anxiety showed a significant positive correlation with the need to temporarily entrust the newborn to the care of medical staff. Women who gave birth via cesarean section and first-time mothers reported an increased need to entrust their newborn to the care of medical staff during the day and at night, and experienced higher levels of pain, fatigue, and anxiety.

Fatigue, pain, and anxiety are symptoms that frequently affect women during the perinatal period. The variety of definitions and measurement methods for these symptoms complicates research in this area. In obstetric literature, pain is described in various contexts, or in connection with anxiety [19,20,21]. In Belgium, labor pain is defined as an unnecessary discomfort that can be alleviated by administering pain relief medication. In contrast, in the Netherlands, labor pain is seen as beneficial in physiological childbirth [21]. There is limited literature examining pain, fatigue, and anxiety among postpartum patients, and how these factors affect their ability to care for the newborn. This study is unique, in that it specifically addresses these interrelated factors within the postpartum period, a time when mothers face significant physical and emotional challenges. By focusing on the relationship between fatigue, pain, and anxiety, and their impact on mother–child bonding and newborn care, this study fills a critical gap in current research. Furthermore, it investigates these issues in a population of women who have experienced a rooming-in birth, providing valuable insights into how this model of care affects maternal wellbeing and caregiving capacity.

However, only a few studies have addressed postpartum fatigue. Fatigue is defined in the Classification of Nursing Diagnoses as “an overwhelming sustained sense of exhaustion and decreased capacity for physical and mental work at usual level” [22]. Fatigue often intensifies as new mothers face numerous challenges and responsibilities, such as caring for the newborn and breastfeeding [23]. Fatigue typically peaks on the third day after childbirth. Recognizing the importance of sleep, it is recommended to educate patients and provide childcare support that does not interfere with exclusive breastfeeding. However, sleep deprivation, excessive fatigue, and breastfeeding place a significant physical strain on the mother, increasing the risk of postpartum depression. Therefore, rooming-in wards should create conditions that allow postpartum women to rest and sleep according to their needs. Support from caregivers and staff in the early days after childbirth plays a crucial role in preventing postpartum depression [24,25]. In women during their early postpartum period, a shorter total sleep time and reduced sleep efficiency are observed, compared to a control group (healthy women who are not pregnant). Additionally, postpartum women show an increase in slow-wave sleep (SWS), likely due to excessive energy expenditure during childbirth [25]. In the postpartum period, many women experience mild mood disturbances, while nearly 15% are diagnosed with postpartum depression within the first 12 months. Additionally, suicide accounts for up to 20% of postpartum deaths [26,27]. Postpartum anxiety is a relatively common phenomenon that can lead to symptoms of anxiety, or even the development of anxiety disorders. As shown by van der Zee-van den Berg et al. [28], as many as 14.7% of women experience anxiety levels, as assessed by the 6-Item State-Trait Anxiety Inventory (STAI-6) scale, at ≥42. The consequences of anxiety disorders affect not only the mother, but also the child. A mother’s anxiety can influence both her interaction with the child and feeding practices, as well as the child’s temperament and socio-emotional development. Additionally, Janssen et al. confirm that prolonged symptoms of anxiety occur more frequently than postpartum depression [29]. Therefore, it is crucial for anxiety assessment to become a routine part of evaluations for women before and after childbirth. According to the International Association for the Study of Pain (IASP) Subcommittee on Taxonomy, pain is defined as “an unpleasant sensory and emotional experience associated with actual or potential tissue damage or described in terms of such damage” [30]. Chronic pain after childbirth may increase the risk of developing postpartum depression, as shown in a study conducted by Zhuang et al. [31]. Therefore, it is essential to manage this pain and minimize the risk of its occurrence.

The rooming-in system is the WHO-recommended practice of care for mothers after birth and their babies. This system has been introduced widely, but its function varies between obstetric centers providing differentiated support for women after childbirth. In the study conducted by Consales et al. [10], the authors confirmed that 48.2% of mothers consistently utilized the rooming-in model. This suggests that more than half of the mothers received support from staff in caring for their infants and periodically entrusted their newborns to staff care. Additionally, the study highlighted solutions suggested by mothers to improve the quality of care in rooming-in units. These included increased staff assistance, organizational and structural changes, and the presence of family members, including overnight stays.8 In the present study, only 34.5% of women were informed about the option of temporarily transferring newborn care to staff.

The need to entrust the baby to the care of the maternal staff was first evaluated by Prokopowicz et al. [12]. In their study, 30% of postpartum women reported the need to temporarily entrust the care of their newborn to staff during the first few days after childbirth [12]. In our own study, 46.1% of respondents expressed the need to entrust their newborn to medical staff during the day, with this need increasing to 53% at night. An important aspect highlighted by Prokopowicz et al. [12] was the higher level of anxiety among women who felt the need to entrust the care of their newborn to staff, compared to those who did not report such a need. The present study further revealed that women who were not informed about the option to temporarily transfer the care of their newborn to staff experienced higher levels of anxiety and fatigue compared to those who received such information.

The need for support in caring for a newborn after childbirth is more commonly observed in women who have undergone cesarean sections, both during the day and at night. This finding aligns with observations reported in both our study and in the study conducted by Prokopowicz et al. [12] Also, Lai et al. [9] demonstrated in their study that women who underwent cesarean sections experienced higher levels of fatigue in the first few days postpartum, compared to those who had vaginal deliveries. They highlighted that this increased fatigue led to greater challenges in performing activities related to newborn care. Rychnovsky et al. [32] reported that as many as 62% of women experienced moderate fatigue, and 18.3% experienced severe fatigue on the second day after childbirth. Zhang et al. [33] indicated that primiparas reported higher levels of anxiety compared to multiparas. In contrast, our study found that primiparas more frequently expressed a need for assistance, which correlates with their elevated anxiety levels.

Identifying limitations in the functioning of maternity wards may help to eliminate barriers that restrict support for postpartum women and contribute to the emergence of negative emotions among them. As emphasized by Munabi-Bibugumira et al. [34], medical staff working in maternity wards encounter significant challenges in delivering high-quality patient care, due to several limitations. These include a lack of essential equipment, supplies, medications, and reliable access to energy and water, as well as insufficient space and facilities within the wards. Additionally, these factors contribute to an increased perception of workload and a higher risk of infections, ultimately diminishing their overall efficiency in care delivery.

Our study found that some obstetric facilities do not offer patients the option of temporary care for their newborns when the mother needs sleep or rest. This option is especially important for women who have had difficult deliveries, as subjectively reported by the patient, such as those who experienced significant blood loss, cesarean sections, operative deliveries, or births during the night, since they often face pain alongside high levels of fatigue and anxiety concerning both their own health and that of their newborns. For mothers to provide safe and nurturing care for their newborns, it is crucial that their basic needs are addressed. In our survey, we asked participants whether they believe postpartum women should have the option to seek support within the rooming-in system. An overwhelming majority of the respondents (92.7%) agreed that postpartum women should have this option.

Psychological birth trauma occurs in 10%–44% of cases. Women who experience this trauma often report psychological distress in subsequent pregnancies, including symptoms such as anxiety, panic attacks, depression, sleep disturbances, and suicidal thoughts [35]. Manurung et al. [36] identified 32 statements from postpartum mothers that can help predict postpartum sadness. Among these, some mothers expressed feelings of being overwhelmed by the continuous care required for their babies in the rooming-in system. Therefore, postpartum care must be optimized and tailored to the physical and psychological condition of the woman after childbirth. The findings from our study align with those of other authors, showing that there are situations in which a woman, due to her physical or emotional condition after childbirth, may be unable to provide continuous, round-the-clock care for her newborn. Additionally, we demonstrated that a mother’s need to entrust the care of her newborn to medical staff is correlated not only with the level of pain, but also with fatigue and anxiety. This situation calls for the implementation of appropriate solutions in rooming-in wards that would allow for temporary care of the newborn by staff or family members around the clock.

Hsiao-Ling Wu suggests that postpartum care centers should provide higher levels of support to mothers in newborn care and dispel myths related to traditional Chinese postpartum confinement practices to promote the rooming-in practice. The authors demonstrated that the willingness of mothers surveyed at a center in southern Taiwan to fully implement the rooming-in practice significantly decreases, among other reasons, due to the stress associated with full-time childcare despite their poor physical condition [37].

Our study has several limitations that should be considered when interpreting the results. The primary limitation is the use of the internet to recruit participants for the survey. This method was chosen because women of reproductive age are generally frequent internet users. However, this may have excluded less educated groups of women, potentially limiting the survey’s representativeness. Another limitation is the potential for recall bias, as women were asked to reflect on their experiences from up to three years ago during their stay in the rooming-in unit. This could lead to inaccuracies in their responses. Additionally, there is the possibility of statistical bias arising from the sampling method and the size of the sample. While the snowball sampling approach may introduce some level of selection bias, the large sample size and robust statistical analysis applied in the study helped mitigate this limitation. The results demonstrated statistically significant correlations that are consistent with findings in the broader literature, supporting the reliability and validity of the chosen methodology. Despite these limitations, the study findings reveal clear trends for improving early postpartum maternal care, aligning with existing research and highlighting important areas for future intervention.

The pilot study is designed to assess the psycho-physical impact of maternal post-partum health problems using simple tools and basic statistical analysis. The approach of keeping the study simple but reliable is commendable, as it recognizes the sensitivity required when working with postpartum populations. By designing an accessible interview that minimizes additional stress for the mothers, this study provides a valuable foundation for future research.

There are known effective approaches to managing pain, such as pharmacotherapy or relaxation techniques, and emotions, such as through providing psychological support. However, in the case of fatigue, the most effective method remains deep sleep, which requires the provision of appropriate conditions and relief in caring for the newborn. Unfortunately, it is not always possible to get help from relatives in this regard. Our study, illustrated in the NEN-opinion figure, clearly shows that mothers feel an urgent need for systemic approaches to regenerate and make up for fatigue deficits.

Deep sleep plays a key role in physical recovery and its deficiency can lead to an accumulation of fatigue. In the context of neonatal care, ensuring that the mother receives an adequate amount of deep sleep is essential to her health and ability to care for her baby. In situations where the support of relatives is not in place, it is necessary to explore alternative systemic approaches that allow mothers to regenerate through adequate sleep [38,39,40,41].

This study provides important insights by exploring the interrelationship between pain, fatigue and anxiety in the postpartum period and their impact on mother–baby bonding and neonatal care. While previous studies have looked at these factors individually, this study is unique in its comprehensive approach to understanding how they together affect a mother’s wellbeing and ability to care. Furthermore, it brings new knowledge by focusing on mothers who have experienced a breech birth, shedding some light on how this model of care affects the mother’s recovery and capacity to care for her newborn, which has been insufficiently explored in the existing literature.

The potential impact of postpartum distress, particularly when amplified by the rooming-in system, warrants careful consideration, due to its association with the development or worsening of medical conditions, such as hypertension, during the peripartum and postpartum periods. This observation underscores the necessity of integrating a multidisciplinary perspective into maternal care. By involving specialists from obstetrics, cardiology, psychiatry, and primary care, healthcare systems can more effectively address the intertwined physical and psychological challenges faced by postpartum women. Such coordinated efforts are essential to improving health outcomes and ensuring a holistic approach to maternal wellbeing [42]. It is acknowledged that incorporating mental health indicators, such as postpartum depression, into the statistical analysis would enhance the study’s scope and relevance. These factors are indeed closely linked to the recovery process and maternal wellbeing, as supported by existing literature [43]. However, as this study represents a pilot analysis, its primary aim was to establish foundational insights into pain, fatigue, and anxiety during the postpartum period.

Additionally, the need for systemic improvements in maternity care, including addressing staff shortages and heavy workloads, is a crucial consideration highlighted by the study findings. The lack of adequate nighttime support for postpartum women underscores the importance of developing targeted protocols to meet these needs effectively. These systemic issues, as discussed in previous studies [40], are essential to improving care outcomes for both mothers and newborns. While this pilot study could not address these factors extensively, they represent an important direction for future research and practical interventions in clinical settings.

## 5. Conclusions

The need to entrust newborns to the care of maternity rooming-in staff is high, but often unmet in many facilities. This need for support in newborn care is correlated with levels of perceived pain, fatigue, and anxiety. Our study found that women often feel overwhelmed by the continuous care required for their babies in the rooming-in system, which can negatively impact the development of the bond between mother and child. To optimize the benefits of rooming-in, healthcare providers should ensure that mothers receive appropriate support, including the option to temporarily hand over care of the newborn to professionals when needed. This approach can help balance the benefits of bonding and breastfeeding with the mother’s need for rest and recovery. Further research should be conducted to explore solutions and plan future actions to alleviate the burdens on postpartum women and facilitate their recovery.

### Clinical Implication

Simple scales to measure pain, fatigue, and anxiety should be implemented as part of the screening process to identify these issues in the early postnatal period. This approach would enable timely clinical interventions, ensuring that mothers do not experience excessive fatigue, and are provided with the option to entrust the care of their newborns when needed. It is essential that this option is framed sensitively to avoid stigmatizing mothers, allowing them to feel supported in their health without experiencing guilt or believing that seeking help is a sign of inadequacy. Such a supportive approach can enhance maternal wellbeing and promote better long-term outcomes for both mothers and their children. This study demonstrates that reliable measurements of pain, fatigue, and anxiety can be achieved using simple 0–10 scales. The implementation of such straightforward tools in clinical practice has the potential to improve care for both mothers and their infants by addressing the key challenges of postpartum wellbeing.

The study utilized the Need to Entrust a Newborn under the Care of the Staff (NEN) scale and the Numerical Rating Scale (NRS) to assess participants’ levels of pain, fatigue, and anxiety. These scales were chosen to provide a comprehensive understanding of the participants’ experiences and needs during the postpartum period. The correlation coefficients derived from these scales highlight the interconnectedness of various aspects of maternal wellbeing, including the significant relationship between fatigue and anxiety. These findings underscore the necessity of addressing these factors together in clinical practice to improve maternal health outcomes. The strong correlation between fatigue and anxiety emphasizes the importance of integrated nursing interventions targeting both physical and psychological symptoms. For instance, structured rest periods could help postpartum women physically recover, while simultaneously alleviating anxiety. Incorporating mental health screenings into routine postnatal care can aid in the early identification of distress, facilitating timely and effective interventions. Psychoeducation programs could further empower mothers by teaching strategies to manage anxiety, such as relaxation techniques, mindfulness practices, and providing access to counseling services.

Clinicians can utilize these findings to optimize care delivery. For example, increasing staff availability during peak hours or at night could ensure that postpartum women receive adequate support for newborn care and personal recovery. Additionally, fostering a calming environment in maternity wards—including providing access to quiet spaces and support from trained professionals—may help mitigate stressors that contribute to fatigue and anxiety. Moreover, these insights highlight the importance of adopting a patient-centered approach that considers the holistic needs of postpartum women. Strategies such as individualized care plans, multidisciplinary collaboration, and evidence-based relaxation techniques can significantly enhance maternal outcomes. Future research should aim to validate these proposed interventions through clinical trials, evaluating their effectiveness in improving maternal recovery and overall wellbeing. Bridging the gap between statistical findings and clinical application remains a critical step toward translating research insights into practical and impactful care strategies.

## Figures and Tables

**Table 1:** Scores for NRS and NEN scales.

Scale	N	Me	Min	Max	IQR
NRS (pain)	1410	6.00	0.00	10.00	5.00
NRS-F (fatigue)	1410	7.00	0.00	10.00	4.00
NRS-A (anxiety)	1410	5.00	0.00	10.00	6.00
NEN-D	1410	3.00	1.00	6.00	3.00
NEN-N	1410	4.00	1.00	6.00	3.00
NEN-opinion	1410	6.00	1.00	6.00	1.00
NEN-information	1410	2.00	1.00	6.00	3.00

IQR, interquartile range; Max, maximum; Me, median; Min, minimum; NRS, Numerical Rating Scale; NEN, Need to Entrust a Newborn under the Care of the Staff scale; D, during the day; N, at night.

**Table 2:** Correlation coefficients between scores of NRS and NEN scales (n = 1410).

Rho Spearman	NEN-D	NEN-N
NRS (pain)	0.296 *	0.332 *
NRS-F (fatigue)	0.423 *	0.485 *
NRS-A (anxiety)	0.422 *	0.431 *

* *p* < 0.001. NRS, Numerical Rating Scale; NEN, Need to Entrust a Newborn under the Care of the Staff scale; D, during the day; N, at night.

**Table 3:** Results for NRS and NEN scales by mode of delivery and number of births.

	VD (n = 810)		CC (n = 600)		U(Z)	Primiparas (n = 840)		Multiparas (n = 570)		U(Z)
	Me	Mrank	Me	Mrank		Me	Mrank	Me	Mrank	
NRS	4.00	572.22	7.00	885.43	−14.361 **	6.00	732.27	5.00	666.06	−3.013 *
NRS-F	7.00	652.13	8.00	777.55	−5.770 **	8.00	764.46	7.00	618.61	−6.660 **
NRS-A	4.00	656.80	5.00	771.25	−5.242 **	6.00	823.08	3.00	532.22	−13.224 **
NEN-D	3.00	648.75	4.00	782.11	−6.178 **	4.00	749.60	3.00	640.52	−5.016 **
NEN-N	3.00	620.81	4.50	819.84	−9.220 **	4.00	751.46	3.00	637.76	-5.228 **

* *p* < 0.01; ** *p* < 0.001. A, anxiety; CC, cesarean section; D, during the day; F, fatigue; Me, median; N, at night; NRS, Numerical Rating Scale; NEN, Need to Entrust a Newborn under the Care of the Staff scale; VD, vaginal delivery.

**Table 4:** NRS, NRS-F, NRS-A scales by comparison in groups of visitors, e.g., presence of visitors (POV) vs. no visitors (NV).

	POV (n = 592)		NV (n = 818)		U(Z)	NEN-I_high (n = 487)		NEN-I_low (n = 923)		U(Z)
	Me	Mrank	Me	Mrank		Me	Mrank	Me	Mrank	
NRS	6.00	693.72	6.00	714.02	−0.929	6.00	678.53	6.00	719.73	−1.816
NRS-F	7.00	708.65	7.00	703.22	−0.249	7.00	621.05	8.00	750.06	−5.708 **
NRS-A	4.00	685.70	5.00	719.83	−1.560	4.00	631.08	5.00	744.76	−5.008 **
NEN-D	3.00	678.61	3.00	724.92	−2.143 *	3.00	667.71	3.00	725.44	−2.572 *
NEN-N	4.00	688.65	4.00	717.69	−1.343	3.00	680.63	4.00	718.62	−1.693
NEN-O	5.50	684.28	6.00	720.86	−1.815	6.00	732.11	6.00	691.46	−1.944

* *p* < 0.01; ** *p* < 0.001.

## Data Availability

Dataset available on request from the authors.

## References

[1] Theo L.O., Drake E. (2017). Rooming-In: Creating a Better Experience. J. Périnat. Educ..

[2] WHO (2009). Baby-Friendly Hospital Initiative: Revised, Updated and Expanded for Integrated Care.

[3] Chien L.-Y., Tai C.-J., Chu K.-H., Ko Y.-L., Chiu Y.-C. (2007). The number of Baby Friendly hospital practices experienced by mothers is positively associated with breastfeeding: A questionnaire survey. Int. J. Nurs. Stud..

[4] Wu H.-L., Lu D.-F., Tsay P.-K. (2022). Rooming-In and Breastfeeding Duration in First-Time Mothers in a Modern Postpartum Care Center. Int. J. Environ. Res. Public Health.

[5] Jaafar S.H., Ho J.J., Lee K.S. (2016). Rooming-in for new mother and infant versus separate care for increasing the duration of breastfeeding. Cochrane Database Syst. Rev..

[6] Andersen L.B., Melvaer L.B., Videbech P., Lamont R.F., Joergensen J.S. (2012). Risk factors for developing post-traumatic stress disorder following childbirth: A systematic review. Acta Obstet. Gynecol. Scand..

[7] Rychnovsky J., Hunter L.P. (2009). The Relationship Between Sleep Characteristics and Fatigue in Healthy Postpartum Women. Women’s Health Issues.

[8] Shiva L., Desai G., Satyanarayana V.A., Venkataram P., Chandra P.S. (2021). Negative Childbirth Experience and Post-traumatic Stress Disorder—A Study Among Postpartum Women in South India. Front. Psychiatry.

[9] Lai Y.-L., Hung C.-H., Stocker J., Chan T.-F., Liu Y. (2015). Postpartum fatigue, baby-care activities, and maternal–infant attachment of vaginal and cesarean births following rooming-in. Appl. Nurs. Res..

[10] Consales A., Crippa B.L., Cerasani J., Morniroli D., Damonte M., Bettinelli M.E., Consonni D., Colombo L., Zanotta L., Bezze E. (2020). Overcoming Rooming-In Barriers: A Survey on Mothers’ Perspectives. Front. Pediatr..

[11] Kurth E., Spichiger E., Stutz E.Z., Biedermann J., Hösli I., Kennedy H.P. (2010). Crying babies, tired mothers—Challenges of the postnatal hospital stay: An interpretive phenomenological study. BMC Pregnancy Childbirth.

[12] Prokopowicz A., Stańczykiewicz B., Uchmanowicz I., Zimmer M. (2022). How to Improve the Care of Women after Childbirth in the Rooming-in Unit: A Prospective Observational Study. Int. J. Environ. Res. Public Health.

[13] Crandall M., Lammers C., Senders C., Savedra M., Braun J.V. (2007). Initial Validation of a Numeric Zero to Ten Scale to Measure Children’s State Anxiety. Anesth. Analg..

[14] Walawender I., Roczniak W., Nowak D., Skowron M., Waliczek M., Rogalska A., Nowak P.G. (2015). Applicability of the Numeric Scale for Anxiety Evaluation in Patients Undergoing Dental Treatment. Dent. Med. Probl..

[15] Karcioglu O., Topacoglu H., Dikme O., Dikme O. (2018). A systematic review of the pain scales in adults: Which to use?. Am. J. Emerg. Med..

[16] Gladman D., Nash P., Goto H., Birt A.J., Lin C.-Y., Orbai A.-M., Kvien T.K. (2020). Fatigue numeric rating scale validity, discrimination and responder definition in patients with psoriatic arthritis. RMD Open.

[17] Hairston I.S., Handelzalts J.E., Lehman-Inbar T., Kovo M. (2019). Mother-infant bonding is not associated with feeding type: A community study sample. BMC Pregnancy Childbirth.

[18] Tzeng Y.-L., Yang Y.-L., Kuo P.-C., Lin Y.-C., Chen S.-L. (2017). Pain, Anxiety, and Fatigue During Labor: A Prospective, Repeated Measures Study. J. Nurs. Res..

[19] Else-Quest N.M., Hyde J.S., Clark R. (2018). Breastfeeding, Bonding, and the mother-Infant Relationship. Merrill Palmer Q..

[20] Curzik D., Jokic-Begic N. (2011). Anxiety sensitivity and anxiety as correlates of expected, experienced and recalled labor pain. J. Psychosom. Obstet. Gynecol..

[21] Christiaens W., Verhaeghe M., Bracke P. (2010). Pain acceptance and personal control in pain relief in two maternity care models: A cross-national comparison of Belgium and the Netherlands. BMC Health Serv. Res..

[22] Zuchatti B.V., Ferreira R.C., Ribeiro E., Duran E.C.M. (2022). Clinical validation of nursing diagnosis fatigue (00093) in women in the immediate hospital postpartum period. Rev. Esc. Enferm. USP.

[23] Senol D.K., Yurdakul M., Ozkan S.A. (2019). The effect of maternal fatigue on breastfeeding. Niger. J. Clin. Pract..

[24] Kawashima A., Detsuka N., Yano R. (2022). Sleep deprivation and fatigue in early postpartum and their association with postpartum depression in primiparas intending to establish breastfeeding. J. Rural Med..

[25] Bei B., Milgrom J., Ericksen J., Trinder J. (2010). Subjective Perception of Sleep, but not its Objective Quality, is Associated with Immediate Postpartum Mood Disturbances in Healthy Women. Sleep.

[26] Payne J.L., Maguire J. (2019). Pathophysiological mechanisms implicated in postpartum depression. Front. Neuroendocr..

[27] Mikšić Š., Uglešić B., Jakab J., Holik D., Srb A.M., Degmečić D. (2020). Positive Effect of Breastfeeding on Child Development, Anxiety, and Postpartum Depression. Int. J. Environ. Res. Public Health.

[28] van der Zee-van den Berg A.I., Boere-Boonekamp M.M., Groothuis-Oudshoorn C.G., Reijneveld S.A. (2021). Postpartum depression and anxiety: A community-based study on risk factors before, during and after pregnancy. J. Affect. Disord..

[29] Janssen A.B., Savory K.A., Garay S.M., Sumption L., Watkins W., Garcia-Martin I., Savory N.A., Ridgway A., Isles A.R., Penketh R. (2018). Persistence of anxiety symptoms after elective caesarean delivery. BJ Psych. Open.

[30] Raja S.N., Carr D.B., Cohen M., Finnerup N.B., Flor H., Gibson S., Keefe F.J., Mogil J.S., Ringkamp M., Sluka K.A. (2020). The revised International Association for the Study of Pain definition of pain: Concepts, challenges, and compromises. Pain.

[31] Zhuang J., Chen Q., Liu C., Zuo R., Zhang Y., Dang J., Wang Z. (2023). Investigating the association between maternal childbirth intention, labor epidural analgesia, and postpartum depression: A prospective cohort study. J. Affect. Disord..

[32] Rychnovsky J.D. (2007). Postpartum Fatigue in the Active-Duty Military Woman. J. Obstet. Gynecol. Neonatal Nurs..

[33] Zhang C.X., Okeke J.C., Levitan R.D., Murphy K.E., Foshay K., Lye S.J., Knight J.A., Matthews S.G. (2022). Evaluating depression and anxiety throughout pregnancy and after birth: Impact of the COVID-19 pandemic. Am. J. Obstet. Gynecol. MFM.

[34] Munabi-Babigumira S., Glenton C., Lewin S., Fretheim A., Nabudere H. (2017). Factors that influence the provision of intrapartum and postnatal care by skilled birth attendants in low- and middle-income countries: A qualitative evidence synthesis. Cochrane Database Syst. Rev..

[35] Pidd D., Newton M., Wilson I., East C. (2023). Optimising maternity care for a subsequent pregnancy after a psychologically traumatic birth: A scoping review. Women Birth.

[36] Manurung S., Setyowati S. (2021). Development and validation of the maternal blues scale through bonding attachments in predicting postpartum blues. Malays. Fam. Physician.

[37] Wu H.-L., Lu D.-F., Tsay P.-K. (2023). Experiences of Implementing Rooming-in Practice for First-Time Mothers in a Postpartum Care Center. SAGE Open Nurs..

[38] Besedovsky L., Lange T., Born J. (2012). Sleep and immune function. Pflügers Arch..

[39] Ackermann S., Rasch B. (2014). Differential Effects of Non-REM and REM Sleep on Memory Consolidation?. Curr. Neurol. Neurosci. Rep..

[40] Baattaiah B.A., Alharbi M.D., Babteen N.M., Al-Maqbool H.M., Babgi F.A., Albatati A.A. (2023). The relationship between fatigue, sleep quality, resilience, and the risk of postpartum depression: An emphasis on maternal mental health. BMC Psychol..

[41] Li H., Li H., Zhong J., Wu Q., Shen L., Tao Z., Zhang H., Song S. (2023). Association between sleep disorders during pregnancy and risk of postpartum depression: A systematic review and meta-analysis. Arch. Womens Ment. Health.

[42] Thomopoulos C., Hitij J.B., De Backer T., Gkaliagkousi E., Kreutz R., Lopez-Sublet M., Marketou M., Mihailidou A.S., Olszanecka A., Pechère-Bertschi A. (2024). Management of hypertensive disorders in pregnancy: A Position Statement of the European Society of Hypertension Working Group ‘Hypertension in Women’. J. Hypertens..

[43] Lee J. (2013). Maternal stress, well-being, and impaired sleep in mothers of children with developmental disabilities: A literature review. Res. Dev. Disabil..

